# Dual Endothelin Antagonism with Aprocitentan as a Novel Therapeutic Approach for Resistant Hypertension

**DOI:** 10.1007/s11906-023-01259-z

**Published:** 2023-08-11

**Authors:** Sayeh Heidari Nejad, Omar Azzam, Markus P. Schlaich

**Affiliations:** 1 Dobney Hypertension Centre, Medical School - Royal Perth Hospital Unit and RPH Research Foundation, The University of Western Australia, Level 3, MRF Building, Rear 50 Murray St, Perth, WA 6000 Australia; 2 Department of Nephrology, Royal Perth Hospital, Perth, Australia; 3 Department of Cardiology, Royal Perth Hospital, Perth, Australia

**Keywords:** Resistant hypertension, Endothelin receptor antagonist, Aprocitentan, Chronic kidney disease, Blood pressure

## Abstract

**Purpose of Review:**

Resistant hypertension (RH) defined as uncontrolled blood pressure despite the use of a combination of a renin-angiotensin system blocker, a calcium channel blocker, and a diuretic at maximally tolerated doses is associated with a substantially increased risk of cardiovascular and renal events. Despite targeting relevant pathophysiological pathways contributing to elevated blood pressure, approximately 10–15% of hypertensive patients remain above recommended blood pressure targets. Further optimization of blood pressure control is particularly challenging in patient populations who frequently present with RH such as elderly and patients with chronic kidney disease, due to the unfavorable safety profile of the recommended fourth-line therapy with mineralocorticoid receptor antagonists. This review explores the potential role of endothelin antagonists as an alternative fourth-line therapy.

**Recent Findings:**

Despite the well-described role of the endothelin pathway in the pathogenesis of hypertension, it is currently not targeted therapeutically. Recently however, main outcome data from the PRECISION study, a randomized placebo-controlled phase 3 trial, in patients with RH on guideline-recommended standardized single-pill background therapy convincingly demonstrated the safety and blood pressure-lowering efficacy of the dual endothelin antagonist Aprocitentan.

**Summary:**

Findings from the phase 3 PRECISION study could signify a turning point in the utilization of endothelin receptor antagonists as a standard treatment for patients with RH.

## Introduction

Hypertension is a prevalent leading cause of disability and mortality worldwide [1]. Resistant hypertension (RH) is a severe form of hypertension which is estimated to affect 10–15% of those patients treated for the condition [2]. RH is defined as blood pressure (BP) above the target range despite being on maximally tolerated doses of at least three antihypertensive drug classes including a blocker of the renin-angiotensin system, a long-acting calcium channel blocker, and a diuretic. Patients who are on four different classes of antihypertensive drugs are considered to have RH regardless of BP measurements [3]. Importantly, RH is associated with higher incidence of hypertension-mediated organ damage and cardiovascular events, such as heart failure, stroke, and chronic kidney disease [4].

With up to 15% of hypertensive patients above recommended BP targets despite the use of three guideline-recommended antihypertensive drugs [5], the necessity of exploring novel therapeutic approaches is obvious. Failure to control BP may indicate that pathways relevant to resistant hypertension are not or not sufficiently opposed by current established approaches. Spironolactone, a mineralocorticoid receptor antagonist, is currently considered the preferred fourth-line therapy of RH following the landmark PATHWAY-2 study demonstrating superior BP lowering efficacy of spironolactone compared to the β-blocker bisoprolol or the α-blocker doxazosin [6]. Spironolactone is not recommended in advanced chronic kidney disease and is commonly associated with development of hyperkalemia and gynecomastia [7, 8].

Currently licensed antihypertensive drug classes are mainly targeting various aspects of the renin–angiotensin–aldosterone system (renin inhibitors, ACE inhibitors, ARBs, Aldosterone antagonists), calcium channels (dihydropyridine and non-dihydropyridine calcium channel blockers), fluid retention and sodium homeostasis (various classes of diuretics), adrenergic receptors (α- and β-blockers), centrally acting sympatholytic agents, and vasodilatory pathways. Targeting the endothelin pathway therapeutically appears as an obvious neglect in the context of RH, given its pivotal role in various aspects of BP control, as summarized below [9••].

## Role of the Endothelin Pathway in Hypertension

**Fig. 1:**
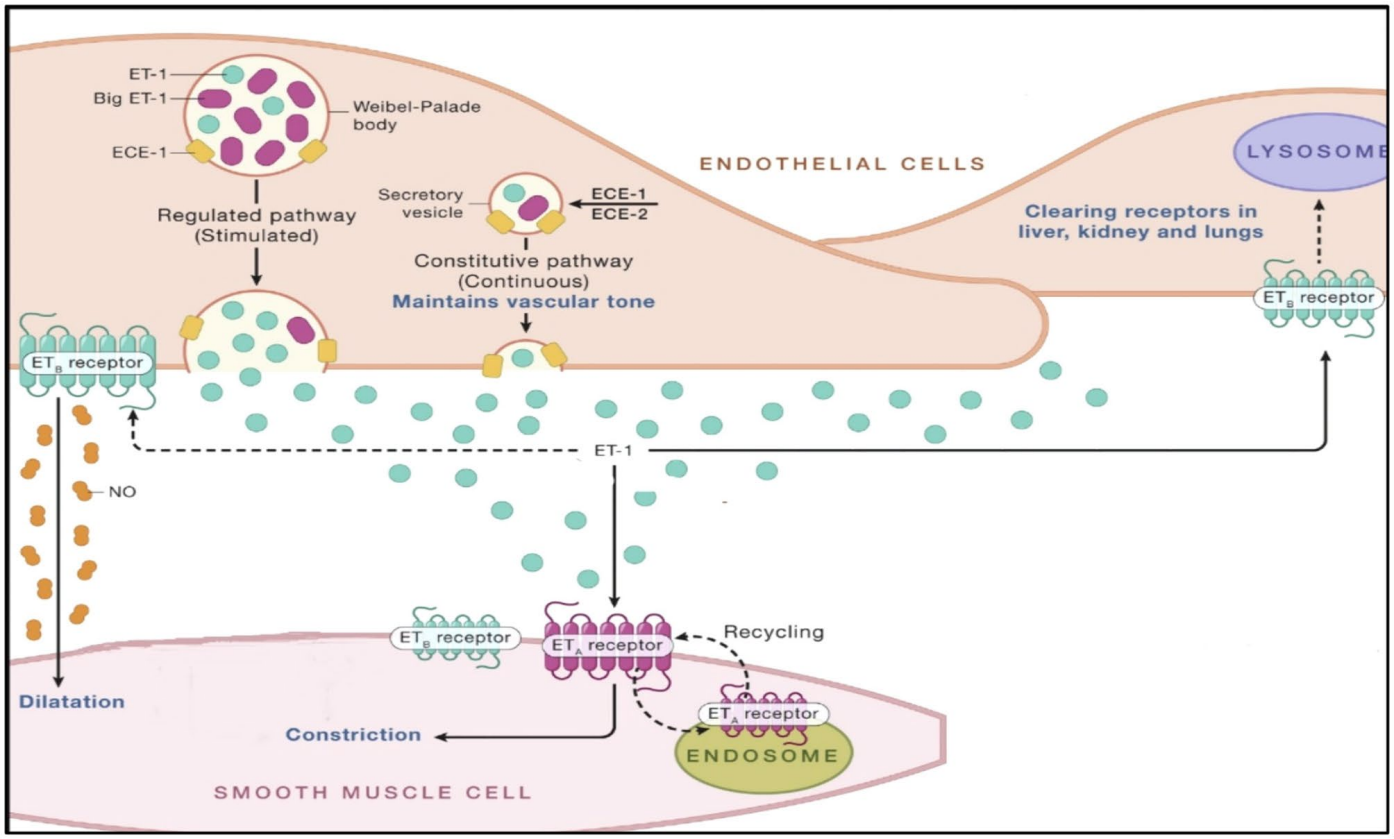
Endothelin pathway in human vasculature (modified with permission from Abraham et al.) [5]

Endothelin-1 (ET-1) is the predominant isoform of the endothelin (ET) family in human cardiovascular system. It is most abundantly produced by vascular endothelial cells both constitutively to maintain vascular tone and in response to different stimuli, while also being generated in a variety of other cells including vascular smooth muscle cells, cardiomyocytes, fibroblasts, macrophages, neurons, and epithelial cells in lungs and kidneys [10, 11•]. In the vasculature, ET-1 acts through two receptors: ET_A_ receptors and ET_B_ (ET_B1_ + ET_B2_) receptors which are located on the vascular smooth muscle cells and endothelial cells regulating BP by inducing vasoconstriction or vasodilation. ET-1 is an extremely potent vasoconstrictor with long half-life, whose primary mechanism of inducing vasoconstriction is via interaction with ET_A_ receptor. In the context of pathophysiological conditions, such as hypertension or pulmonary hypertension, the interaction between ET-1 and ET_B2_ in the smooth muscle cells also leads to vasoconstriction. Vasodilation by ET-1 is mediated by ET_B1_ on endothelial cells via production of NO and PGI_2_ (Fig. 1).

ET_B_-mediated paracrine release of NO and other vasodilators is crucial in acting as a counterregulatory pathway to limit ET_A_-mediated vasoconstriction. In pathophysiological conditions where there is endothelial dysfunction with a loss of vasodilators, the vasoconstrictor and other pathophysiological effects of ET-1, such as cell proliferation, inflammation and fibrosis, will be potentiated [12].

Overexpression of both tissue ET-1 and ET receptors is shown in various pathologies including essential hypertension, pulmonary arterial hypertension, chronic kidney disease, and diabetes mellitus, which has led to the development of multiple selective ET_A_R and mixed ET_A_R and ET_B_R antagonists over the years [5, 10, 11•]. Although there is significant evidence supporting the involvement of ET-1 in the pathophysiology of hypertension [13], clinical trials have not yet convincingly demonstrated the efficacy of ET receptor antagonists as a treatment for systemic or resistant hypertension. In this review, we provide an overview of relevant studies in the context of human hypertension and explore how these findings and recent results from the PRECISION trial are likely to shape the future role of endothelin receptor antagonists in modern hypertension management.

## Development and History of Endothelin Receptor Antagonists (ERAs)

Krum et al. [14] conducted the first human study in 1998 to evaluate the short-term effect of bosentan, a combined ET_A_R and ET_B_R antagonist, on mild-to-moderate essential hypertension. Eligible patients were selected based on their age (≥ 18 years) and manually measured average office sitting mean diastolic pressure of 95–115 mmHg as well as a mean diastolic pressure higher than 85 mmHg on 24-h ambulatory blood pressure monitoring (ABPM).

**Fig. 2:**
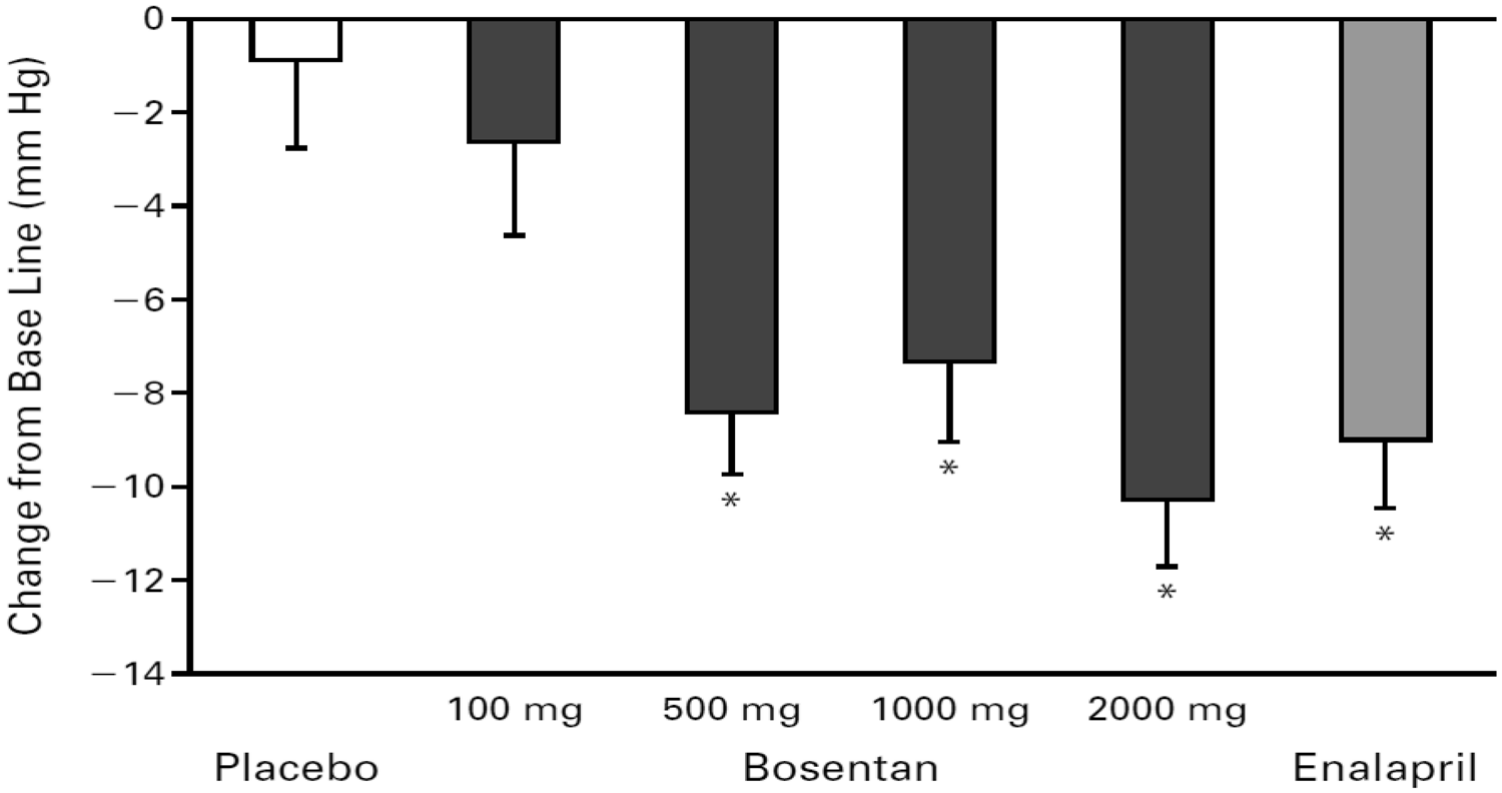
Mean (± SE) change from baseline in office systolic blood pressure in patients with mild-to-moderate hypertension assigned to receive placebo, bosentan (100, 500, 1000, or 2000 mg daily), or enalapril (20 mg daily) (with permission from [14])

Out of the 511 screened patients, 293 were randomly assigned to one of the six treatment groups, including placebo and different daily doses of bosentan and enalapril. Results showed that bosentan administration resulted in a significant reduction in both office diastolic and systolic pressure compared to placebo (Fig. 2), and the reduction in blood pressure with a daily dose of 500 mg or more of bosentan was similar to that observed with enalapril. However, some adverse events were reported with bosentan, including headache, flushing, leg edema, and asymptomatic increases in serum alanine and aspartate aminotransferase levels, with higher incidence in the 2000 mg bosentan dose group, raising initial concerns regarding hepatic safety.

In 2002, Nakov et al. [15] conducted a multicenter randomized, double-blind clinical trial to evaluate the efficacy of Darusentan, a selective ET_A_R antagonist, in patients with moderate HTN, defined as resting diastolic office BP in sitting position between 100 and 109 mmHg at baseline. Patients with secondary causes of HTN or significant cardiovascular complications in the prior 6 months were excluded from the study. Three hundred ninety-two patients received placebo or Darusentan at varying doses (10, 30, or 100 mg) for 6 weeks, followed by a 2-week placebo withdrawal period. The mean of three readings taken in the sitting position at 2-min intervals after 10 min of rest was used to measure automated office BP (AOBP) at baseline and following visits.

Compared to a previous study [10] of bosentan, Darusentan demonstrated a significant dose-dependent reduction in both diastolic and systolic BP with unchanged pulse rate, starting from the first week of double-blind treatment, which continued throughout the next 5 weeks. At the end of the placebo withdrawal period, patients who had previously received Darusentan experienced increased BP. In patients with stage 1 or 2 hypertension, Darusentan, when used as a single agent at a dose of 100 mg daily, reduced BP by approximately 11/8 mmHg, after correcting for the placebo response, after 6 weeks of treatment.

Inadequate BP reduction and adverse events led to discontinuation of the study by 29.6% of randomized patients. The adverse event profile of the 10- and 30-mg Darusentan groups was comparable to placebo, while it was higher in the 100-mg Darusentan group. Headache, flushing, and peripheral edema were the most reported adverse events.

This study suggested that ET_A_R antagonism could be a promising new therapeutic approach for hypertension treatment; however, endothelin receptor antagonists should be used only in specific patients, such as those with RH due to potential side effects.

Indeed, such a study was subsequently conducted by Weber et al. [16] investigating the effectiveness of varying doses of Darusentan in reducing BP in patients with resistant hypertension (RH). RH was defined as having a systolic BP of 140 mmHg or more while being treated with at least three BP-lowering drugs, including a diuretic, at full or maximum tolerated doses [17]. The study also included patients with comorbidities such as diabetes mellitus, heart disease, and chronic kidney disease to better represent the general population of RH patients in clinical practice.

The study included a 2-week single-blind run-in period to stabilize BP on background antihypertensive medications. Three hundred seventy-nine patients were randomly assigned to receive either placebo (*n* = 132) or Darusentan at doses of 50 mg (*n* = 81), 100 mg (*n* = 81), or 300 mg (*n* = 85) daily for 14 weeks. Most patients’ background antihypertensive medications consisted of blockers of the renin-angiotensin system (97%), calcium channel blockers (74%), and beta-blockers (66%), in addition to a diuretic (99%), which was primarily a thiazide or frusemide.

**Fig. 3:**
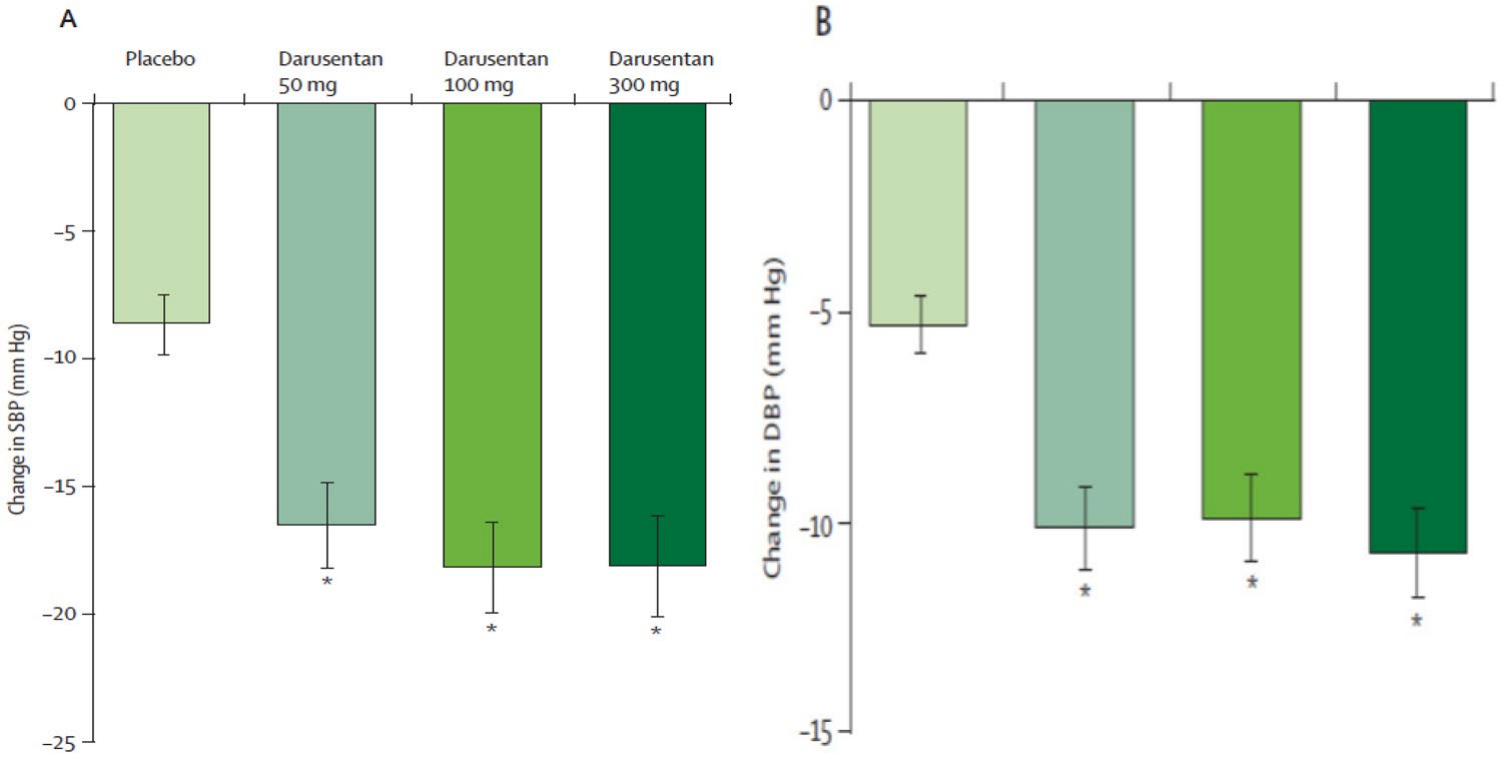
Changes from baseline in clinic seated blood pressure after 14 weeks of treatment with placebo or Darusentan 50, 100, or 300mg. **A** Change in systolic blood pressure (SBP). **B** Change in diastolic blood pressure (DBP). Error bars show SE. *p < 0.0001 (with permission from [16])

**Fig. 4:**
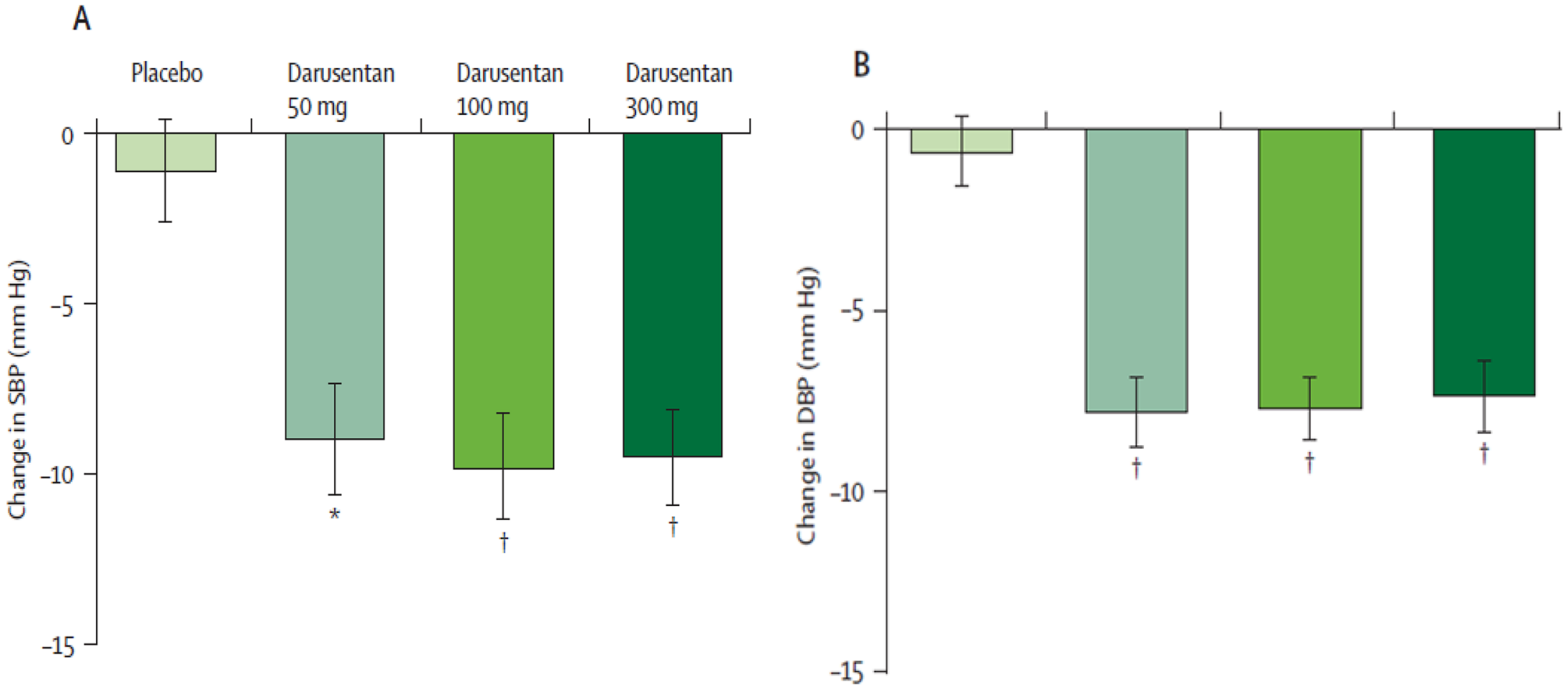
Changes from baseline in mean 24-h ambulatory blood pressure after 14 weeks. **A** Change in systolic blood pressure (SBP). **B** Change in diastolic blood pressure (DBP). Error bars show SE. *p = 0.0002. †*p* < 0.0001 (with permission from [16])

The study found that Darusentan significantly reduced mean clinic systolic and diastolic BP compared to placebo as shown in Fig. 3, and the reductions were consistent with result from ambulatory blood pressure monitoring (Fig. 4). Interestingly, there was no dose–response relationship for Darusentan over the 50–300-mg dose range, suggesting that using higher doses may not provide additional clinical benefit. Darusentan could effectively reduce clinic blood pressure by an additional 10 mmHg in patients with RH already receiving multiple antihypertensive drugs at recommended doses. The drug was also more likely to achieve the recommended goal for systolic BP in patients with diabetes (< 140 mmHg) and chronic kidney disease (< 130 mmHg), who commonly present with RH. Fluid retention due to vasodilation was observed in 27% of patients receiving Darusentan, which could exacerbate heart failure. This effect was mostly reported during the initial 6 weeks of therapy, indicating that monitoring patients during the early stages of treatment and adjusting diuretic therapy can manage this issue. Only two patients receiving Darusentan reported elevated liver transaminase values, and there was a modest decrease in estimated GFR (3–6 mL/min/1.73 m^2^) in patients receiving higher doses of Darusentan. Urinary albumin excretion was reduced by about 60% in the combined Darusentan groups.

In 2010, the second randomized double-blind placebo-controlled study was conducted to evaluate the effect of Darusentan in RH compared to placebo or the central 2 agonist guanfacine [18]. The study included 849 RH patients who were receiving at least three antihypertensive drugs, including a diuretic. The patients were randomized to receive either Darusentan (dosed at 50, 100, or 300 mg), placebo, or guanfacine (1 mg) orally, daily for 14 weeks.

**Fig. 5:**
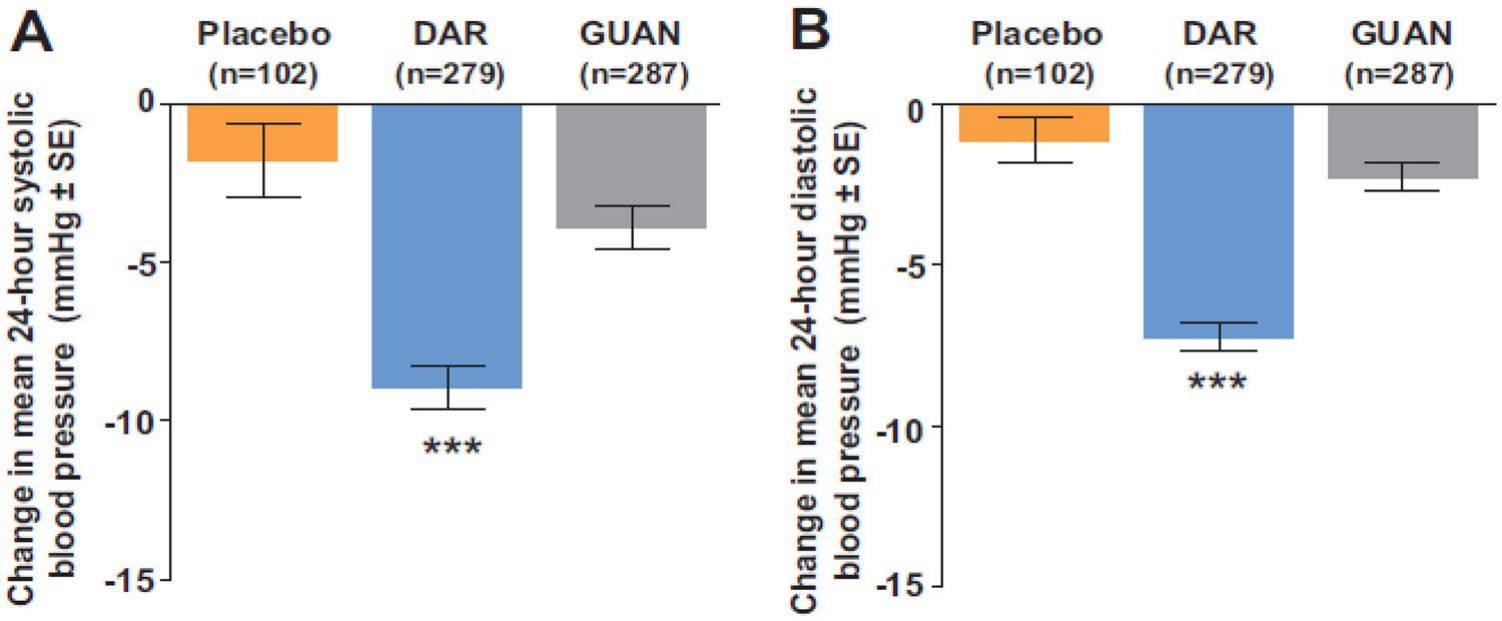
Mean changes from baseline in 24-h ambulatory BP after 14 weeks. Only patients with both baseline and week 14 ambulatory readings were included in this analysis. ***p < 0.001 as compared with placebo or guanfacine (with permission from [18])

Unlike the first trial of Darusentan in RH [16], this study failed to demonstrate a significant difference between the Darusentan and placebo groups in terms of change in clinic systolic BP from baseline; however, Darusentan led to greater reductions in 24-h ambulatory systolic and diastolic BP compared to either placebo or guanfacine which was identical to those seen in previous trial (Fig. 5) [16]. This lack of difference was primarily due to a large placebo response observed late (> 8 week) in the trial (− 14/ − 8 mmHg) that could not be explained. If the primary end point of this study had been chosen as changes in ambulatory BP, the results of the study may have been interpreted in a different manner. The primary adverse effects observed in the Darusentan group were edema and/or fluid retention, occurring mainly during the first 6 weeks after the start of treatment, resulting in heart failure exacerbation in one patient.

The results of the 24-h ambulatory BP analysis of this trial along with previous trials suggested that ET_A_ antagonists could be a useful additional therapeutic approach to better manage RH, but concerns remain about the risk of fluid retention and worsening heart failure symptoms despite diuretic therapy in those already at high risk. The overall balance of benefits and risks, including long-term outcomes, of this class of drugs for treating RH had yet to be determined.

## The PRECISION Study

**Fig. 6:**
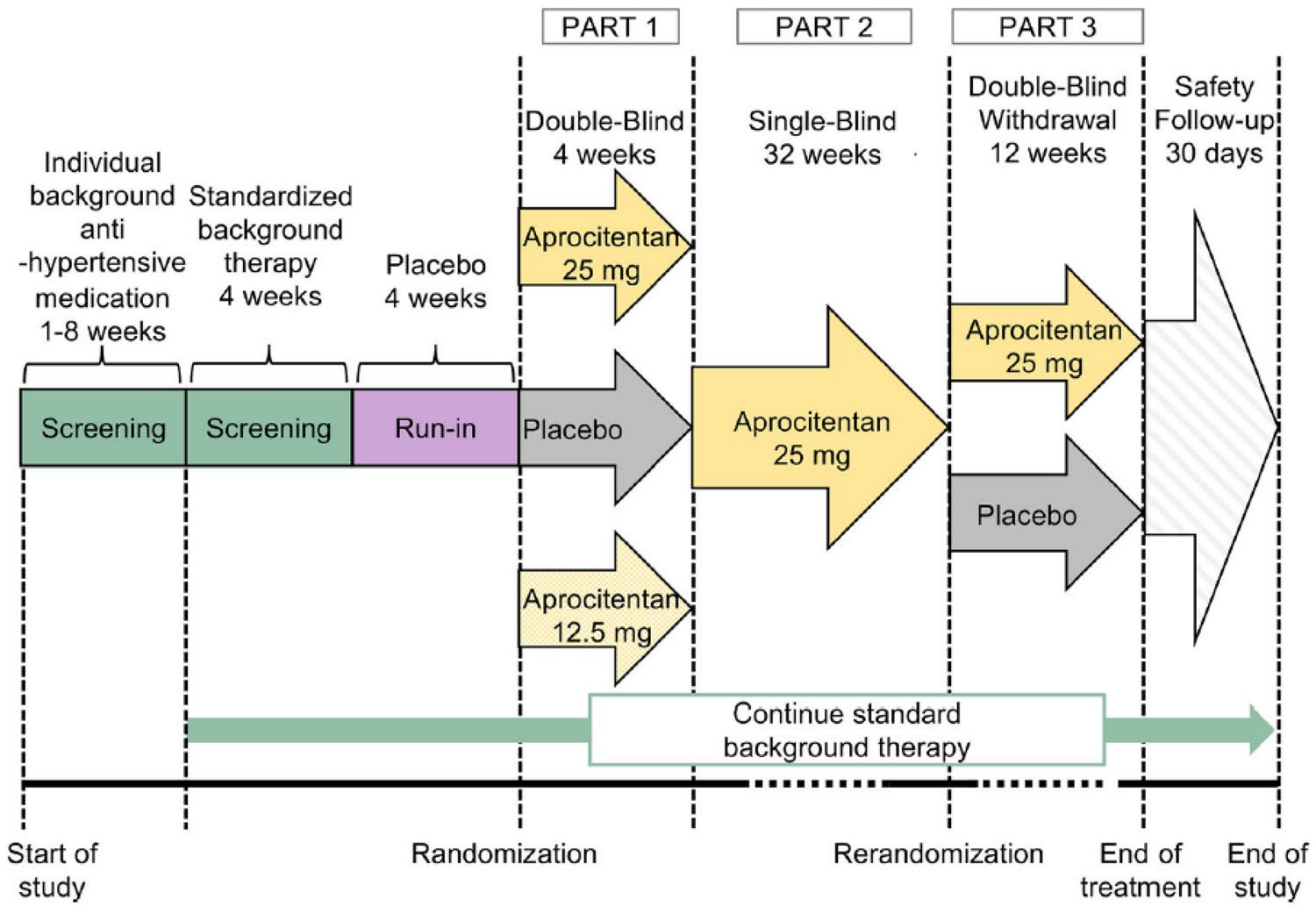
PRECISION study design

The most recent study to address the potential utility of ET receptor antagonists in RH was PRECISION, a phase 3 multicenter, blinded, randomized, and parallel group trial [9••]. In contrast to previous studies, PRECISION used a potent dual ET_A_/ET_B_ receptor antagonist, Aprocitentan, which has a longer half-life and has been shown to have a favorable safety profile [19, 20]. It does not cause hepatotoxicity and appears to result in less fluid retention due to its dual blockade effect on endothelin receptors. It was also suggested to have synergistic effect on BP combining with renin–angiotensin–aldosterone system inhibitors or calcium channel blockers in animal studies [20].The primary goal of the study was to evaluate the safety and antihypertensive effect of Aprocitentan, administered in conjunction with three different classes of antihypertensive medications, compared to placebo in patients with RH. The unique design of the PRECISION study consisted of three major parts, which adequately addressed various deficiencies present in earlier studies (Fig. 6).

In the initial stage of the study, all adults who had a history of RH—defined as a sitting systolic BP (siSBP) of at least 140 mmHg, as measured by unattended automated office blood pressure (uAOBP), after taking at least three different classes of antihypertensive medications for a minimum of 4 weeks—were subjected to screening. The aim was to increase the sensitivity of diagnosis and treatment efficacy by rigorously excluding secondary causes of hypertension and adopting a higher uAOBP threshold (systolic BP  ≥ 140 mmHg) for RH definition. In contrast to previous studies, the PRECISION study also recruited patients who are representative of the population commonly affected by resistant hypertension and its associated complications, such as those with diabetes, cardiovascular disease, cerebrovascular disease, and chronic kidney disease.

Thereafter, all eligible patients were transitioned to a standardized background therapy (SBT) for weeks. This SBT included maximally tolerated doses of a single-pill combination of a calcium channel blocker (amlodipine), an angiotensin receptor blocker (valsartan), and a diuretic (hydrochlorothiazide) at fixed doses of either 5/160/25 mg or 10/160/25 mg. To minimize the placebo response, a 4-week single-blind (SB) run-in phase was incorporated, during which patients received a placebo in conjunction with SBT.

The second stage of study involved a sequential approach comprising a double-blind (DB) randomized phase (4 weeks) with patients receiving placebo, Aprocitentan 12.5 mg, or Aprocitentan 25 mg in a 1:1:1 ratio; an extended single-blind (SB) phase (32 weeks) where all patients received Aprocitentan 25mg; and a double-blind–withdrawal (DB-WD) phase (12 weeks) where patients were re-randomized to either Aprocitentan 25 mg or placebo in a 1:1 ratio. This allowed for a comprehensive assessment of the continued antihypertensive efficacy of Aprocitentan over a substantial period. This was followed by a 30-day safety follow-up period. Throughout the study, participants were assessed using a 24-h ambulatory BP monitoring at week 4 and week 40 and through mean uAOBP at each visit, and adverse events were recorded. A notable advantage of the study was the thorough monitoring of medication adherence, a key problem in patients treated for HTN [21] during the entire study duration. This was accomplished through various approaches, including pill counting, observation of pill consumption before ambulatory BP monitoring, and examination of urine samples to detect background medication intake.

**Fig. 7:**
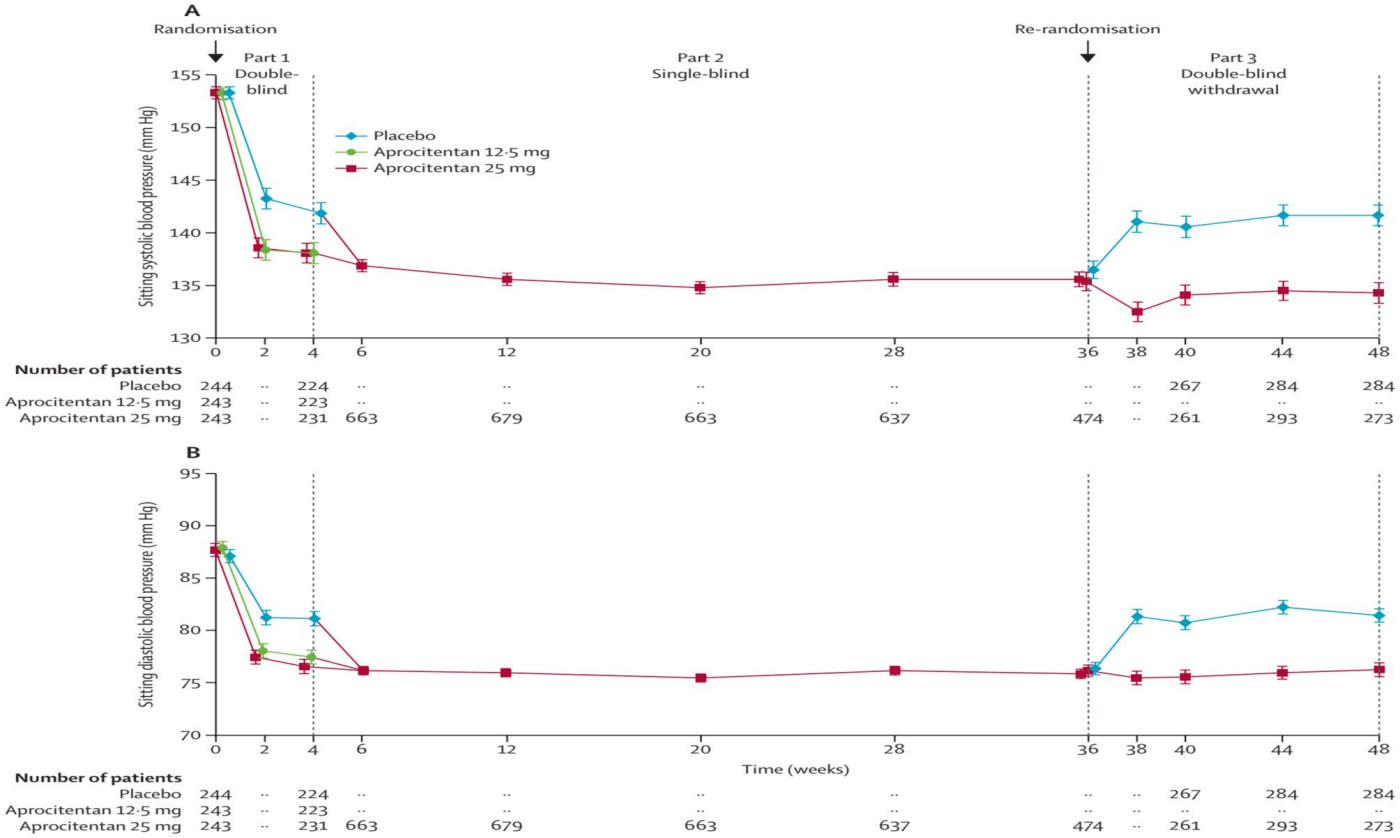
Sitting systolic and diastolic BP measured as unattended automated office BP over time (with permission from [9••])

Out of 1965 screened participants, 911 were included in the placebo run-in period, and 730 were randomized. The most common reason for exclusion (44.4% of 1965) was failure to meet the SiSBP ≥ 140 mmHg before randomization. Figure 7 summarizes the short-term (4 weeks) and long-term (up to 48 weeks) blood pressure-lowering effects of Aprocitentan, as reflected in changes in systolic and diastolic uAOBP. After 4 weeks, both Aprocitentan doses (12.5 mg and 25 mg) resulted in a greater reduction in office SBP than placebo, with reductions of − 15.3 mmHg and − 15.2 mmHg, respectively, compared to − 11.5 mmHg for placebo. A decrease in office DBP was also observed for both Aprocitentan doses, with reductions of − 3.9 mmHg and − 4.5 mmHg, respectively, compared to placebo. During the second part of the study, patients previously on Aprocitentan maintained their blood pressure reductions, while those previously on placebo experienced a further BP reduction which was maintained throughout the 32 week single-blind phase. During part 3, when re-randomisation to either placebo or Aprocitentan 25mg occurred at week 36, BP was maintained with Aprocitentan 25mg whereas BP increased with placebo. The difference in office systolic and diastolic BP remained significant between the Aprocitentan and placebo groups up to week 48.

At week 4, a significant decline in SBP was observed for older patients (aged ≥ 75 years), patients with macro-albuminuria (urine albumin-creatinine ratio > 300 mg/g), and those with chronic kidney disease stage 3–4 (estimated glomerular filtration rate [eGFR] 15 to < 60 mL/min per 1·73 m2). The clinical benefit is particularly significant given the higher cardiovascular risk profile of the indicated study groups. No difference in treatment effect was detected between patients with or without β-blocker treatment at screening.

**Fig. 8:**
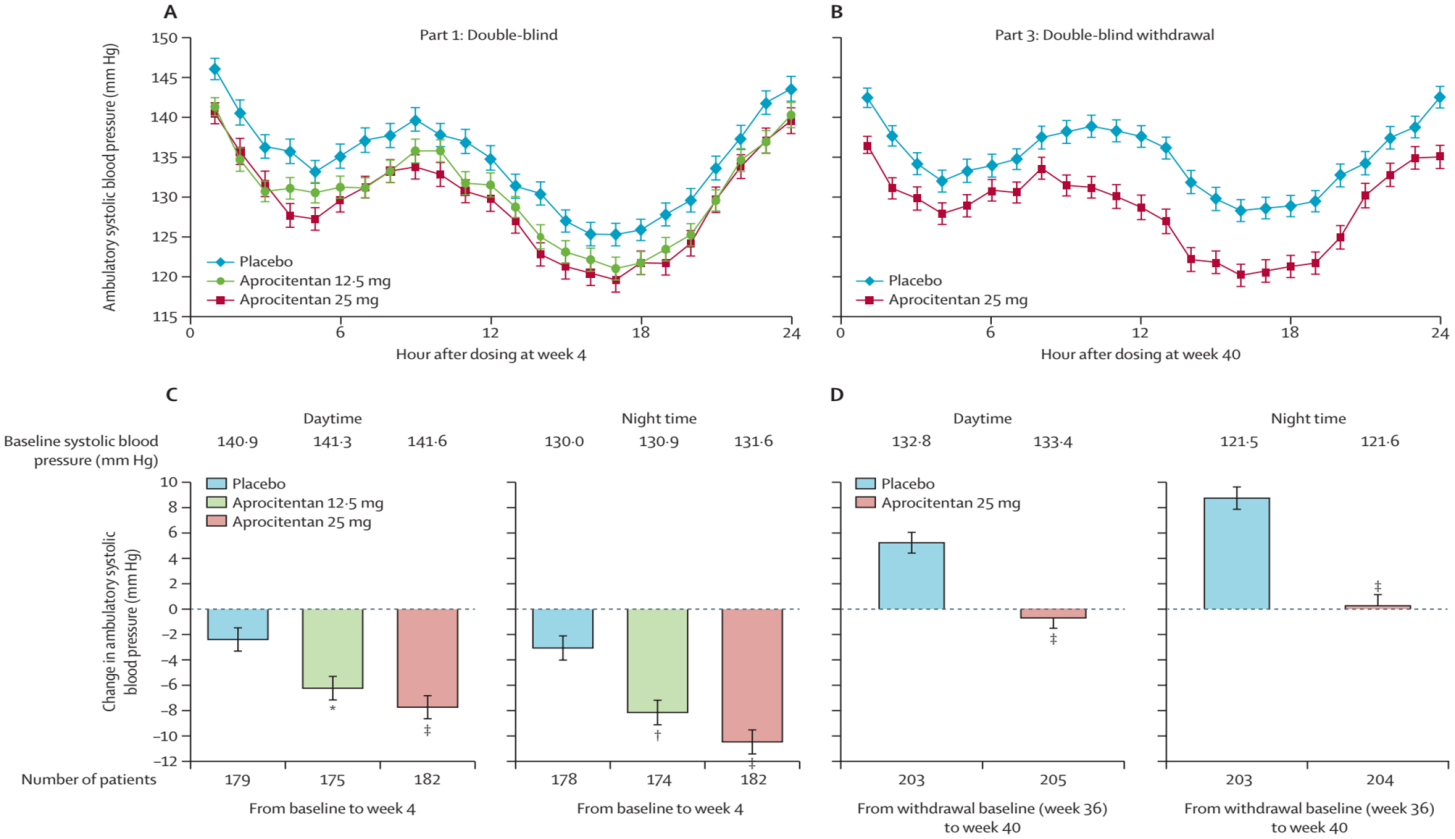
Systolic BP measured by 24-h ambulatory BP monitoring after dosing and corresponding least square mean changes in daytime and nighttime ambulatory BP from baseline to week 4 and week 40 (with permission from [9••])

The results from ABPM provided additional verification of the office readings (Fig. 8). A significant decline was observed in the BP levels during the night, which is a more accurate predictor of cardiovascular mortality compared to other BP indices [22, 23].

As anticipated, Aprocitentan was associated with mild-to-moderate fluid retention compared to placebo in a dose-dependent manner (9.1%, 18.4%, and 2.1% for patients receiving Aprocitentan 12.5 mg, 25 mg, and placebo, respectively, during the 4-week part 1). Edema was managed with addition or uptitration of diuretic therapy. Seven patients taking Aprocitentan 25mg discontinued the study during parts 1–3 due to edema, and 10 out of 11 patients who required hospitalization for nonfatal heart failure were taking Aprocitentan during parts 1–3. However, all patients had a high cardiovascular risk and relevant medical history including pre-existing heart failure, chronic kidney disease, and diabetes. A slight increase in N-terminal pro-brain natriuretic peptide (NT-proBNP) and mid-regional pro-atrial natriuretic peptide (MR-proANP) was observed in part 1 with Aprocitentan, followed by stabilization during part 2 and reversal during part 3. There was no evidence of hepatotoxicity.

## Putting Recent Endothelin Antagonist Studies Into Context

### Implications of the Available Evidence on ET Receptor Antagonists and Hypertension

The unopposed endothelin pathway might represent an important contributor to persistent RH in patients on current pharmacological therapies targeting the three well-established pathophysiologic pathways. As demonstrated by the PATHWAY-2 study, the mineralocorticoid receptor antagonist spironolactone is currently recommended as the preferred fourth-line therapy option for RH where suitable, showing a decrease in home systolic BP by 8.7 mmHg after 12 weeks of treatment. Common adverse effects of spironolactone include hyperkalemia and reduction in eGFR which limits the use of this medication in high-risk patients frequently presenting with RH such as those with stage 3 and 4 chronic kidney disease. Higher doses of spironolactone can lead to gynecomastia and erectile dysfunction, particularly in higher doses of 50 mg or more per day for primary resistant hypertension. The PRECISION study demonstrated that Aprocitentan could be a particularly useful treatment option for patients who are unsuitable for spironolactone, such as the elderly and those with chronic kidney disease stage 3–4. Indeed, a greater reduction in SBP was observed during the fourth week of study among these specific patient groups, i.e., older patients aged ≥ 75 years, as well as those with macro-albuminuria (urine albumin-creatinine ratio > 300 mg/g) and chronic kidney disease stage 3–4 [9••].

Furthermore, Aprocitentan may exhibit a synergistic effect on BP reduction when co-administered with conventional medications by neutralizing the effect of endothelin pressor system, which is distinct from the renin-angiotensin aldosterone pathway and its impacts on blood vessels and kidneys [20].

Proteinuria is an independent risk factor for chronic kidney disease progression and cardiovascular events. Aprocitentan has demonstrated substantial antiproteinuric properties, especially in patients with chronic kidney disease stage 3–4 with RH. The Study of Diabetic Nephropathy with Atrasentan (SONAR), showed that atrasentan, a selective ET_A_ receptor antagonist, reduced urine albumin-creatinine ratio by 51.8% in patients with type 2 diabetes and CKD (eGFR of 25–75 mL/min per 1·73 m^2^ of body surface area) after a 6-week treatment. Moreover, long-term use of endothelin receptor antagonists substantially reduced the risk of end-stage kidney disease in high-risk patients with type 2 diabetes [24]. These findings suggest that ET receptor antagonists have the potential to protect renal function and reduce cardiovascular risk in patients with moderate-to-severe chronic kidney disease, in addition to their blood pressure-lowering effect. Exciting new opportunities may exist by combining ET receptor antagonists with potentially complementary treatments such as SGLT2 inhibitors to further reduce proteinuria, provide greater renal protection, and mitigate the potential of fluid retention. In the ongoing ZENITH-CKD trial, a phase 2b multicenter study that aims to evaluate the efficacy and tolerability of combination therapy with dapagliflozin and zibotentan, a potent ET_A_ receptor antagonist, compared to dapagliflozin alone, in patients with chronic kidney disease and albuminuria will explore the potential of such an approach (Zenith-CKD trial ClinicalTrials.gov Identifier: NCT04724837).

The most common adverse effect related to ET receptor antagonists is mild-to-moderate fluid retention occurring in a dose-dependent manner predominantly in the first 4–6 weeks of therapy. To enhance patient compliance and prevent exacerbation of pre-existing heart failure, it seems essential to maintain a euvolemic state by prescribing appropriate diuretics prior to commencing therapy. Patient’s volume status should be closely monitored particularly during the first 4–6 weeks of treatment. Reassuringly, ET receptor antagonist associated fluid retention is very responsive to additional diuretic therapy, particularly loop diuretics and therefore clinically manageable.

The PRECISION study has the potential to revolutionize the common application of ET receptor antagonists. PRECISION provided substantial evidence for a new treatment option for patients with high cardiovascular risk and RH, who have not responded to current therapies, by effectively targeting the unopposed endothelin pathway with a favorable safety profile. The pronounced impact on nocturnal blood pressure, known to be the most accurate predictor of cardiovascular death, and the favorable effects in elderly and in patients with chronic kidney disease, patient cohorts in whom BP control is known to be particularly difficult to achieve, render Aprocitentan a very useful addition to the therapeutic armamentarium for resistant hypertension.

## Data Availability

Not applicable.

## References

[1] Collaborators GBDRF. Global, regional, and national comparative risk assessment of 84 behavioural, environmental and occupational, and metabolic risks or clusters of risks, 1990–2016: a systematic analysis for the Global Burden of Disease Study 2016. Lancet. 2017;390(10100):1345–422. 10.1016/S0140-6736(17)32366-8 PMC5614451 28919119

[2] Doumas M, Imprialos KP, Kallistratos MS, Manolis AJ. Recent advances in understanding and managing resistant/refractory hypertension. F1000Res. 2020;9. 10.12688/f1000research.21669.1 PMC7065661 32201574

[3] Williams B, Mancia G, Spiering W, Rosei EA, Azizi M, Burnier M, et al. [2018 ESC/ESH guidelines for the management of arterial hypertension. The Task Force for the management of arterial hypertension of the European Society of Cardiology (ESC) and the European Society of Hypertension (ESH)]. G Ital Cardiol (Rome). 2018;19(11 Suppl 1):3S-73S. 10.1714/3026.30245 30520455

[4] Sim JJ, Bhandari SK, Shi J, Reynolds K, Calhoun DA, Kalantar-Zadeh K (2015). Comparative risk of renal, cardiovascular, and mortality outcomes in controlled, uncontrolled resistant, and nonresistant hypertension. Kidney Int.

[5] Abraham GR, Davenport AP (2023). From ABCD to E for endothelin in resistant hypertension. Cell..

[6] Williams B, MacDonald TM, Morant S, Webb DJ, Sever P, McInnes G (2015). Spironolactone versus placebo, bisoprolol, and doxazosin to determine the optimal treatment for drug-resistant hypertension (PATHWAY-2): a randomised, double-blind, crossover trial. Lancet.

[7] Chapman N, Dobson J, Wilson S, Dahlof B, Sever PS, Wedel H (2007). Effect of spironolactone on blood pressure in subjects with resistant hypertension. Hypertension.

[8] Pitt B, Zannad F, Remme WJ, Cody R, Castaigne A, Perez A, et al. The effect of spironolactone on morbidity and mortality in patients with severe heart failure. Randomized Aldactone Evaluation Study Investigators. N Engl J Med. 1999;341(10):709–17. 10.1056/NEJM199909023411001 10471456

[9] Schlaich MP, Bellet M, Weber MA, Danaietash P, Bakris GL, Flack JM (2022). Dual endothelin antagonist aprocitentan for resistant hypertension (PRECISION): a multicentre, blinded, randomised, parallel-group, phase 3 trial. Lancet.

[10] Dhaun N, Goddard J, Kohan DE, Pollock DM, Schiffrin EL, Webb DJ (2008). Role of endothelin-1 in clinical hypertension: 20 years on. Hypertension.

[11] Dhaun N, Webb DJ (2019). Endothelins in cardiovascular biology and therapeutics. Nat Rev Cardiol.

[12] Iglarz M, Binkert C, Morrison K, Fischli W, Gatfield J, Treiber A (2008). Pharmacology of macitentan, an orally active tissue-targeting dual endothelin receptor antagonist. J Pharmacol Exp Ther.

[13] Xu M, Lu YP, Hasan AA, Hocher B (2017). Plasma ET-1 concentrations are elevated in patients with hypertension meta-analysis of clinical studies. Kidney Blood Press R.

[14] Krum H, Viskoper RJ, Lacourciere Y, Budde M, Charlon V, Investigators BH (1998). The effect of an endothelin-receptor antagonist, bosentan, on blood pressure in patients with essential hypertension. New Engl J Med.

[15] Nakov R, Pfarr E, Eberle S, Investigators H (2002). Darusentan: an effective endothelin A receptor antagonist for treatment of hypertension. Am J Hypertens.

[16] Weber MA, Black H, Bakris G, Krum H, Linas S, Weiss R (2009). A selective endothelin-receptor antagonist to reduce blood pressure in patients with treatment-resistant hypertension: a randomised, double-blind, placebo-controlled trial. Lancet.

[17] Lenfant C, Chobanian AV, Jones DW, Roccella EJ (2003). Seventh report of the Joint National Committee on the prevention, detection, evaluation, and treatment of high blood pressure (JNC 7) resetting the hypertension sails. Hypertension.

[18] Bakris GL, Lindholm LH, Black HR, Krum H, Linas S, Linseman JV (2010). Divergent results using clinic and ambulatory blood pressures: report of a darusentan-resistant hypertension trial. Hypertension.

[19] McCoy EK, Lisenby KM (2021). Aprocitentan (a dual endothelin-receptor antagonist) for treatment-resistant hypertension. J Cardiovasc Pharmacol.

[20] Trensz F, Bortolamiol C, Kramberg M, Wanner D, Hadana H, Rey M (2019). Pharmacological characterization of aprocitentan, a dual endothelin receptor antagonist, alone and in combination with blockers of the renin angiotensin system, in two models of experimental hypertension. J Pharmacol Exp Ther.

[21] Carey RM, Calhoun DA, Bakris GL, Brook RD, Daugherty SL, Dennison-Himmelfarb CR (2018). Resistant hypertension: detection, evaluation, and management: a scientific statement from the American Heart Association. Hypertension.

[22] Dolan E, Stanton A, Thijs L, Hinedi K, Atkins N, McClory S (2005). Superiority of ambulatory over clinic blood pressure measurement in predicting mortality - the Dublin Outcome Study. Hypertension.

[23] Cardoso CRL, Salles GC, Salles GF (2020). Prognostic importance of on-treatment clinic and ambulatory blood pressures in resistant hypertension a cohort study. Hypertension.

[24] Heerspink HJL, Parving HH, Andress DL, Bakris G, Correa-Rotter R, Hou FF (2019). Atrasentan and renal events in patients with type 2 diabetes and chronic kidney disease (SONAR): a double-blind, randomised, placebo-controlled trial. Lancet.

